# A Review of Demographic, Medical, and Treatment Variables Associated with Health-Related Quality of Life (HRQOL) in Survivors of Hematopoietic Stem Cell (HSCT) and Bone Marrow Transplantation (BMT) during Childhood

**DOI:** 10.3389/fpsyg.2017.00253

**Published:** 2017-03-10

**Authors:** Trude Reinfjell, Marta Tremolada, Lonnie K. Zeltzer

**Affiliations:** ^1^Department of Psychology, Norwegian University of Science and TechnologyTrondheim, Norway; ^2^Department of Child and Adolescent Psychiatry, St. Olavs University HospitalTrondheim, Norway; ^3^Department of Developmental and Social Psychology, University of PaduaPadua, Italy; ^4^Department of Pediatrics, David Geffen School of Medicine at UCLALos Angeles, CA, USA

**Keywords:** childhood cancer, survivors, hematopoietic stem cell transplant, bone marrow transplant, health-related quality of life, pain

## Abstract

Hematopoietic stem cell transplantation (HSCT) is a standard treatment after disease relapse and failure of conventional treatments for cancer in childhood or as a first line treatment for some high-risk cancers. Since hematopoietic stem cells can be found in the marrow (previously called a bone marrow transplantation) or periphery, we refer to HSCT as inclusive of HSCT regardless of the origin of the stem cells. HSCT is associated with adverse side effects, prolonged hospitalization, and isolation. Previous studies have shown that survivors of HSCT are at particularly high risk for developing late effects and medical complications, and thus, in addition to survival, quality of life in survivors of HSCT is an important outcome. This review summarizes and distills findings on the health-related quality of life (HRQOL) of long-term childhood cancer survivors of HSCT and examines significant sociodemographic, medical, disease and treatment correlates of HRQOL, as well as the methodology of the studies (instruments, type of studies, timing of assessment, type of transplantation). Because previous reviews covered the studies published before 2006, this review searched three databases published between January, 2006, and August, 2016. The search identified nine studies, including 2 prospective cohort studies and 7 cross-sectional studies. All studies reported a follow-up time of >5 years. The review found that HRQOL is significantly impacted over time following childhood HSCT, with salient correlates of HRQOL found to be presence of a severe chronic health or major medical condition, graft vs. host disease (GVHD), or pain. Continual evaluation of HRQOL must be integrated into long-term follow-up after childhood HSCT, and intervention should be offered for those survivors with poor HRQOL. Longitudinal studies should be emphasized in future research to allow for predictor models of resilience and poor HRQOL.

## Introduction

New treatments for childhood cancer have resulted in significant improvements in 5-year survival rates to 80% for most children (Phillips et al., 2015). It has been estimated that 62% of adult survivors of childhood malignancy have ≥1 and 38% ≥2 treatment-induced chronic health conditions, and 28% have a severe or life-threatening problem, especially survivors of CNS tumors or hematopoietic stem cell transplantation (HSCT) (Oeffinger et al., 2006; Diller et al., 2009). Advancements in HSCT have contributed to a dramatic increase in pediatric cancer survival rates (Clarke et al., 2008). Many children and adolescents undergo HSCT after disease relapse and failure of conventional treatments and are vulnerable because HSCT is typically associated with significant adverse side effects, prolonged hospitalization, isolation and a high mortality rate 1 year post-transplant (Barrera et al., 2006).

Survivors of HSCT are at risk for late effect complications, with >90% suffering from at least one and >70% from at least three chronic conditions (Bhatia et al., 2011). Typical risk factors related to HSCT include: (1) patients conditioned with total brain irradiation (TBI), (2) higher-dose conditioning chemotherapy, (3) treatment before transplant, (4) development of serious complication after HSCT, (5) potentially toxic supportive care drugs, and (6) chronic graft vs. host disease (GVHD) (Bhatia et al., 2011). Various physical symptoms, such as pain, nausea, and fatigue, further worsen physical dysfunction (Lowe et al., 2007; Armenian et al., 2011).

In summary, previous studies have highlighted chronic health conditions found in survivors of childhood HSCT, with those with GVHD being particularly vulnerable. A study of marrow-derived HSCT, irrespective of GVHD status (active or resolved), found a high burden of multiple (≥2) health conditions (Armenian et al., 2011), that continue to increase with longer-term follow-up (Sun et al., 2010).

However, health is defined as not merely the absence of disease but rather a state of complete physical, mental, and social well-being. Health-related quality of life (HRQOL) is the most frequently used approach in epidemiological and clinical health research to assess and monitor children's overall QOL (Wallander and Koot, 2016). HRQOL is a multidimensionality construct covering physical, emotional, mental, social and behavioral components of well-being and functioning as perceived by patients and/or other observers (Ravens-Sieberer et al., 2006). During the last decade, there have been 4 reviews of the HRQOL literature on survivors after childhood HSCT. All concluding that most survivors have a HRQOL comparable to that of healthy controls, but there are subgroups that are more vulnerable to negative impact on HRQOL (Tsimicalis et al., 2005; Clarke et al., 2008; Packman et al., 2010; Tanzi, 2011). However, the limited longitudinal studies question the validity of this conclusion (Tsimicalis et al., 2005; Clarke et al., 2008). In previous studies, HRQOL was examined before and up to 6 or 12 months post-HSCT (Clarke et al., 2008; Packman et al., 2010). In addition, a study by Barrera et al. (2009) assessed HRQOL pre-HSCT and up to 2 years post-HSCT. These prospective studies suggest that HRQOL improves over a 4- to 12- and 24-month period post-transplant. Parsons et al. (2012) emphasized the lack of studies beyond 5 years post-transplant, even though the majority of pediatric HSCT recipients are long-term survivors.

We conducted this review to determine how HSCT in childhood may affect survivors' HRQOL later in life, with a specific focus on studies with a long-term focus published in the last decade. In reviewing studies of HRQOL in long-term childhood cancer survivors, we also assessed the significant sociodemographic, health and medical, disease, and treatment correlates of HRQOL and the methodology of the studies (instruments, type of studies, timing of assessment, type of transplantation).

## Materials and methods

The online databases Medline (OVID), PubMed, and PsycINFO were searched using the following terms: hematopoietic stem cell transplant, bone marrow transplant, child, childhood, children, adolescent, young adults, survivor, cancer, late effect, health-related quality of life, quality of life, psychosocial, psychological, health, pain. This literature search was limited to papers published between January 2006 and August 2016. The inclusion criteria were the following: papers in English in a peer-reviewed journal, at least one formal standardized HRQOL measure, age 21 or younger at transplant, HSCT for malignancy or hematological disease, HRQOL as an outcome measure, and follow-up ≥5 years' post-transplant. We excluded literature reviews, case studies, or studies involving purely qualitative methodology.

## Results

### Study characteristics

We identified 9 studies based on the eligibility criteria. Two were prospective cohort studies measuring HRQOL up to a mean time of 13.5 years post-HSCT (Berbis et al., 2013; Schultz et al., 2014). Seven cross-sectional studies were identified, and four of the nine were multi-center studies (Michel et al., 2007; Berbis et al., 2013; Schultz et al., 2014; Kenzik et al., 2015).

All studies, except the study by Kenzik et al. (2015), used comparisons of outcome with one of the following population norms: US norms (Forinder et al., 2006), age-matched norms (Lof et al., 2009), a gender- and age-matched control cohort (Sanders et al., 2010), other cancer diagnosis/treatment groups (Michel et al., 2007; Sundberg et al., 2010; Clarke et al., 2011; Schultz et al., 2014), or both norms and a cancer group (Berbis et al., 2013). Sample sizes ranged from 18 to 662 (Kenzik et al., 2015). The mean age at assessments was 8–42 years for cross-sectional studies and 19.6–20 years for longitudinal, prospective research. See Table 1.

**Table 1:** **The long-term impact of HSCT in childhood cancer survivors on HRQOL**.

**Study**	**Cases (N), Age at time of evaluation (years)**	**Sample, Comparison group, Transplant type**	**Time since diagnosis to evaluation**	**Informant, HRQOL instrument, Pain measurements**	**Results**	**Variables associated with outcome**
**LONGITUDINAL**						
Berbis et al. (2013), Multi center (5), France	*N* = 256 Mean ± SD: 19.6 ± 7.1	HSCT Group *N* = 256 (27%) Chemotherapy group (ALL, AML) *N* = 687 (treated with conventional therapy) Age- and gender-matched French norms Allogeneic transplantation n = 191 (74.6%)	Mean, SD: 12.5 ± 6.4	Survivors self-report SF-36 VSP-A Parents VSP-Ap	1. HSCT adult survivors have lower HRQOL (physical, bodily pain, general health perceptions) compared with the conventional therapy group. 2. Transplanted survivors with cGVHD had a higher risk of late effects and reported more altered QOL. 3. The survivors as a group reported lower mental composite scores compared with French norms but no significant differences for the physical composite score. 4. For child survivors, there were no significant differences between the treatment groups based on parent reports.	1. GVHD
Schultz et al. (2014), Multi center (4), US	*N* = 180 Median (range): 20 (8-39)	Chemotherapy group (AML) *N* = 180 Chemotherapy followed by auto-BMT = 26 Chemotherapy followed by allo-BMT = 54 Chemotherapy only = 100	Median, (range): 13.5, (6–22)	Survivors Self-report SF-36 Cancer-related pain (CCSS baseline questionnaire)	1. HRQOL scores were similar among the treatment groups. 2. Lower physical HRQOL was observed in survivors reporting more health conditions or cancer-related pain. 3. Severe chronic health conditions predicted physical but not mental HRQOL. 4. Among allo-BMT survivors, the presence of a severe chronic health condition was associated with a lower physical mean summary score.	1. Severe chronic health conditions. 2. Presence of a major medical condition. 3. Cancer-related pain.
**CROSS-SECTIONAL**						
Sundberg et al. (2013), Single center, Sweden	*N* = 18 Median (range): 27 (18–37)	Survivors of lymphoblastic malignancy *N* = 70 Treated with SCT, *N* = 18 Treated without SCT (survivors of childhood ALL), *N* = 52 Autologous stem cells *n* = 15 Allogeneic *n* = 3	Median, (range): 18, (10–22)	Survivors self-report SEIQoL-DW SF-36	1. Poorer overall QOL and more negative consequences were related to dysfunctions in the HSCT group. 2. Being unemployed or on sick leave were associated with a decline in HRQOL and individual QOL.	1. Unemployment or on sick leave.
Clarke et al. (2011), Multi center (4), UK	N=29 Mean (SD), (range): 13.79 (2.62), (10-18)	HSCT *N* = 29 Non-HSCT *N* = 25 UK norms Allogeneic transplant *n* = 29	Mean, (range): 5.09, (1–14)	Survivors self-report Mother's proxy report *n* = 29 PedsQL4.0 Mother's own mental health SF-12 Teachers n= 14	1. HRQOL scores for the HSCT group were significantly lower in all domains compared with the non-transplanted group and population norms, but were not significantly related to clinical variables. 2. Children with more late effects (problems with vision, growth, weight and fertility) had lower HRQOL scores reported by mothers. 3. Mothers in the HSCT group had significantly poorer mental well-being than population norms.	
Forinder et al. (2006), Single center, Sweden	*N* = 42 Mean, (range): 8 (9–18)	Leukemia or myelodysplastic syndromes 60%, *n* = 25, Non-malignancy (*n* = 17) US norms Allogeneic SCT *n* = 25	Mean, (range): 8, (3-20)	Survivors self-report SCHQ – CF87 Parent-proxy report SCHQ – PF50 Child Health Questionnaire Pain measured by the Subjective health and symptom inventory checklist	1. Overall HRQOL comparable with norms, but bodily pain was higher, and general health and self-esteem were lower. 2. Symptom severity and late effects (including GVHD) were associated with lower HRQOL in post HSCT survivors. 3. Parents reported lower psychosocial and physical HRQOL for their children.	1. GVHD
Michel et al. (2007) Multi center (2), France	*N* = 142 Mean, (range): 18.6, (8–18)	Leukemia (ALL; 69% *n* = 98; AML; 31%, *n* = 44) No HSCT *N* = 288, Pediatric cancer survivors (chemotherapy group) Allogeneic transplant *n* = 92 Autologous transplant *n* = 50	Mean, (range): 11.9, (6-18)	Survivors self-report SF-36 VSP-A Parents VSP-Ap	1. In adults, there were significant differences in the subscale “General Health” and the Physical Composite Score, with the HSCT group reporting lower scores than the chemotherapy group, but the effect sizes were less than 0.2. 2. Similar HRQOL for children and adolescents in both groups. 3. Transplanted survivors had more side-effects (height growth failure, gonadal dysfunction, hypothyroidism and cataract).	1. Physical adverse effect.
Lof et al. (2009), Single center, Sweden	*N* = 53 Mean, (*SD*), (range): 26 (5.8), (18–42)	Malignant disorders, *n* = 35 (67%) Non-malignant disorders, *n* = 18 (33%) Age-matched norms Allogenic: (match), sibling *n* = 45, parent *n* = 2, and heterologous (unrelated donor) *n* = 6 TBI=33 (62%)	Mean, (range): 17, (5–28)	Survivors self-report SWED-QUAL Pain measured by the Subjective health and symptom inventory checklist	1. Physical health HRQOL was reported to be poorer post HSCT than age-matched norms, whereas emotional well-being was similar to that of norms. 2. HSCT group reported poorer HRQOL within sexuality than age-matched norms. 3. In total, 53% reported pain, and 42% had more memory and concentration problems. 4. Older age, time elapsed post SCT and fewer self-reported symptoms correlated with better HRQOL.	
Sanders et al. (2010) Single center, US	*N* = 214 Mean, range: 28.7 (18.8–45.9) Gender- and age-matched control cohort	Myeloid malignancy *n* = 68, Lymphoid malignancy *n* = 69, CML *n* = 24 (leukemia *n* = 148) (69%), Non-malignant disease (31%) 21% received 18.0-24.0 Gy cranial irradiation Autologous *n* = 8 Allogenic (match) *n* = 48 Heterologous (mismatch) *n* = 12	Mean, (range): 16.2,(5.2-28.9)	Survivors self-report SF-36	1. Physical functioning worse in transplant patients. 2. Myeloid malignancy was a significant risk factor, as was having received an autologous transplant. 3. Females had worse functioning than males.	Diagnosis: 1. Myeloid malignancy 2. Female 3. >18 years post- transplant
(Kenzik et al., 2015) Multi center study (40), North America	*N* = 662 Mean, (*SD)*, (range): 42.1 (11) (18–71) No comparison group	Most participants had a low severity of treatment experience (60%) and a less intense previous treatment (66%) Low-autologous no GVHD *n* = 390 Moderate-allogeneic no GVHD *n* = 168 High-allogeneic GVHD n=88	Mean, (range): 7.0, (1.8–22)	Survivors self-report SF-36	1. Physical symptoms were the most strongly significant factor in poor physical HRQOL. 2.Depressive symptoms impacted mental HRQOL more than physical HRQOL.	1. Physical symptoms most strongly associated with physical HRQOL.

*HSCT, hematopoietic stem cell transplant; BMT, bone marrow transplant; auto-BMT, autologous bone marrow transplant; allo-BMT, allogeneic bone marrow transplant; ALL, acute lymphoblastic leukemia; AML, acute myeloid leukemia; CML, chronic myelogenous leukemia; GVHD, chronic graft vs. host disease; HRQOL, health-related quality of life; QOL, quality of life; SF-36, Medical Outcomes Study 36-item Short Form Health Survey; SEIQOL-DW, The Schedule for the Evaluation of Individual Quality of Life; SWED-QUAL, The Swedish HRQOL profile; PedsQL4.0, Pediatric Quality of Life Measure; VSP-A, Vécu et Santé Percue de l‘Adolescent et de l’ enfant; SCHQ - CF87, Child Health Questionnaire - Child Form; SCHQ - PF50, Child Health Questionnaire - Parent Form; TBI, Total Body Irradiation, CCSS, Childhood Cancer Survivors Study*.

Regarding transplant type, four studies included patients undergoing either allogeneic or autologous transplant (Michel et al., 2007; Sanders et al., 2010; Sundberg et al., 2010; Schultz et al., 2014; Kenzik et al., 2015), while three studies included allogeneic patients only (Forinder et al., 2006; Lof et al., 2009; Clarke et al., 2011; Berbis et al., 2013). Treatment included HSCT with either marrow- or peripherally-derived stem cells (Forinder et al., 2006; Michel et al., 2007; Lof et al., 2009; Sanders et al., 2010; Sundberg et al., 2010; Clarke et al., 2011; Berbis et al., 2013; Schultz et al., 2014; Kenzik et al., 2015).

Standardized HRQOL measures were used in all studies, including 6 different measures (Table 1). The Medical Outcomes Study 36-Item Short-Form Health Survey (SF-36) (Reulen et al., 2006) was used in six studies as a measure of HRQOL (Michel et al., 2007; Sanders et al., 2010; Berbis et al., 2013; Sundberg et al., 2013; Schultz et al., 2014; Kenzik et al., 2015). One study used the Pediatric Quality of Life Measure (PedsQL 4.0) (Clarke et al., 2011). Other QOL measurements included: SEIQoL-DW (Sundberg et al., 2013), Child Health Questionnaire (SCHQ-CF87) (Forinder et al., 2006), and The Swedish HRQOL profile (SWED-QUAL) (Lof et al., 2009). Five studies used survivors' self-reports (Lof et al., 2009; Sanders et al., 2010; Sundberg et al., 2013; Schultz et al., 2014; Kenzik et al., 2015), four studies used both survivors' self-reports and parent proxy reports (Forinder et al., 2006; Michel et al., 2007; Clarke et al., 2011; Berbis et al., 2013), and one study used survivors' self-reports and teacher reports (Clarke et al., 2011). Seven studies used generic HRQOL measures (Michel et al., 2007; Lof et al., 2009; Sanders et al., 2010; Clarke et al., 2011; Berbis et al., 2013; Schultz et al., 2014; Kenzik et al., 2015), and two studies used both a generic and a disease-related measure (Forinder et al., 2006; Sundberg et al., 2013).

Symptoms of pain in relation to HRQOL were identified in three studies (Forinder et al., 2006; Lof et al., 2009; Schultz et al., 2014), and GVHD was assessed in relation to HRQOL in three studies (Forinder et al., 2006; Lof et al., 2009; Berbis et al., 2013). A subjective health and symptom inventory checklist, including a pain scale score and measures of GVHD and late effects, was used in two studies (Forinder et al., 2006; Lof et al., 2009). The pain scale included items relating to pain severity, whereas the subjective symptom scale included items relating to GVHD and late effects. Schultz et al. (2014) measured cancer-related pain (from the Childhood Cancer Survivors Study (CCSS) baseline questionnaire) in addition to using the SF-36. Berbis et al. (2013) measured GVHD, dividing the HSCT group into two subgroups with or without post-transplantation chronic graft-vs.-host disease (cGVHD), and compared health status and QOL in each of them with the conventional chemotherapy group. In most studies (Michel et al., 2007; Sanders et al., 2010; Berbis et al., 2013; Sundberg et al., 2013; Schultz et al., 2014; Kenzik et al., 2015), pain was measured as part of the SF-36; a separate standardized pain measure was adopted only in three studies (Forinder et al., 2006; Lof et al., 2009; Schultz et al., 2014).

### The relationship between childhood HSCT and long-term HRQOL

Contradictory findings were reported in the two longitudinal studies (Berbis et al., 2013; Schultz et al., 2014). In Berbis et al. (2013), childhood HSCT adult survivors 12.5 years post-diagnosis reported lower HRQOL in physical domains, bodily pain, and general health perceptions compared with the conventional therapy group. The survivors, as a group, also reported lower mental composite scores compared to French norms. However, Schultz et al. (2014) found HRQOL scores to be similar among treatment groups 13.5 years post-diagnosis. A cross-sectional study by Michel et al. (2007) found significant differences in the subscale “General health” and in the Physical Composite Score 11.9 years post-diagnosis. The HSCT group reported lower scores than the chemotherapy group, but the effect sizes were less than 0.2, suggesting uncertain clinical significance. The cross-sectional study by Sundberg et al. (2013), with a mean follow-up time of 18 years, also found poorer overall QOL and more negative outcomes in the HSCT group. Similarly, the study by Clarke et al. (2011) found HRQOL scores for the HSCT group to be significantly lower in all domains compared with both the non-transplanted group and population norms. In contrast to those studies, the study by Forinder et al. (2006) showed that HRQOL 8 years post-diagnosis was overall comparable with norms, although bodily pain was higher and general self-esteem lower, and parents reported lower psychosocial and physical HRQOL for their children. However, Lof et al. (2009) found 17 years post-diagnosis that physical HRQOL was reported to be poorer post-HSCT compared with age-matched norms. Similarly, Sanders et al. (2010) found physical functioning to be worse in the transplanted patients 16.2 years post-diagnosis compared with a gender-and age-matched control cohort.

### Variables associated with HRQOL in long-term survivors of childhood HSCT

Variables associated with poor HRQOL in long-term adult survivors of childhood HSCT based on multivariate analyses, included unemployment or being on sick leave (Sundberg et al., 2013), female gender (Sanders et al., 2010), >18 years after transplant (Sanders et al., 2010), physical symptoms (Kenzik et al., 2015), a severe chronic health condition or major medical condition (Michel et al., 2007; Schultz et al., 2014), myeloid malignancy diagnosis (Sanders et al., 2010), cancer-related pain (Schultz et al., 2014), and GVHD (Forinder et al., 2006; Berbis et al., 2013). Sanders et al. (2010) found more time elapsed post-HSCT to be associated with poorer HRQOL.

## Discussion

This review investigated HRQOL in long-term survivors of childhood HSCT. Most studies identified compromised HRQOL compared with conventional therapy groups and population norms (Forinder et al., 2006; Michel et al., 2007; Lof et al., 2009; Sanders et al., 2010; Clarke et al., 2011; Berbis et al., 2013; Sundberg et al., 2013). This finding is in contrast to a previous review by Clarke et al. (2008), who noted improvement in HRQOL 12–24 months post-transplant. This discrepancy suggests that a to-be-determined minimum amount of years are necessary to detect late effects on HRQOL. The studies included in the present review had a minimum follow-up time since diagnosis of 5 years; however, the mean follow-up time for several studies was greater than 18 years.

Factors associated with long-term HRQOL include unemployment or being on sick leave, time since HSCT, physical symptoms, a severe chronic health condition/major medical condition, cancer-related pain, and GVHD. Previous studies have reported contradictory results regarding correlates of HRQOL and psychological symptom outcomes. Childhood HSCT survivors overall were found to have more medical adverse effects or HSCT-related complications (Lee et al., 2006), that was strongly associated with worse HRQOL. Two studies found an early reduction in HRQOL immediately after allogeneic HSCT but a consistent recovery to baseline levels thereafter (Notteghem et al., 2003; Barrera et al., 2009). However, Clarke et al. (2008) emphasized that, although HRQOL is within the baseline range 1 year post-transplant, accumulating evidence suggests that the pre-transplant baseline is seriously compromised by the disease for which the patient is being transplanted. In this regard, the severity of the disease prior to transplantation (e.g., myeloid malignancy) may infer a higher likelihood of poor HRQOL after transplantation (Sanders et al., 2010).

Baker and Fraser (2008) indicate that the strongest association between reduced HRQOL and impaired functional status following HSCT is the presence of chronic GVHD, which can negatively impact physical and mental health and lead to the development of functional impairment and activity limitations over a lifetime (Lee et al., 2006). Research on pediatric HSCT focusing on GVHD is limited. Very long-term follow-up is important and large-scale studies are lacking (Bhatia et al., 2011; Parsons et al., 2012). In the present review, only the longitudinal study by Berbis et al. (2013) found that childhood HSCT survivors with GVHD had a higher association with late effects and reported greater reduction in HRQOL than survivors without GVHD. In that study, 74.6% of 256 HSCT survivors had allogeneic HSCT.

Only three studies have had a specific focus on pain and its relationship with HRQOL. In a previous study, allogeneic HSCT recipients with active chronic GVHD had the strongest association between poorer HRQOL and poor general health, functional impairment, and pain (because of cancer or its treatment) compared with CCSS survivors without GVHD (Armenian et al., 2011). However, most of the studies in the present review only measured pain using the two pain items in the SF-36. Only one study by Schultz et al. (2014) also included self-reported cancer-related pain (CCSS–baseline study), and the studies by Forinder et al. (2006) and Lof et al. (2009) included a pain scale measured by the Subjective Health and Symptom Inventory checklist. In the present review, Forinder et al. (2006) reported an association between HRQOL among childhood HSCT survivors and bodily pain, general health, and self-esteem. Schultz et al. (2014) found that, among allogeneic HSCT survivors (28% with a history of GVHD), the presence of a severe chronic health condition was associated with a lower physical mean summary score; however, these survivors were no more likely to report cancer-related pain or cancer-related anxiety than comparison cohorts. Overall HRQOL scores were similar among the treatment groups, although survivors reporting more health conditions or cancer-related pain were more likely to have diminished HRQOL. These results are similar to those in the Sanders et al. (2010) study. However, these findings suggest that complications and diminished physical functioning may be problematic outcomes of acute myeloid leukemia (AML) vs. acute lymphoblastic leukemia (ALL) survivors of HSCT. Mulrooney et al. (2008), reporting from the CCSS, emphasized that both chronic fatigue and pain as long-term outcomes should be further investigated.

There is a need for longer-term longitudinal studies in childhood HSCT survivors as late effects may not be visible until later in life. Unfortunately, only two longitudinal studies with a mean follow-up time of 12.5 (Berbis et al., 2013) and 13.5 years (Schultz et al., 2014) were found. The other seven were cross-sectional.

The low number of research studies found for this review limits strong conclusions, as cross-sectional data in the majority prevent the estimation of causal relationships among variables. Statistical power is usually not stated or insufficient to detect significance, effect-sizes are often not reported when results are significant, and the four single-center studies lack generalizability to other treatment sites. Further, more ethnically diverse samples would lend greater generalizability to the findings. Recruitment bias needs to be considered any long-term outcome studies since survivors with diminished HRQOL may be less likely to participate. The single study by Schultz et al. (2014) examining recruitment bias found no demographic or medical differences between participants and non-participants treated on identical protocols. Children with cancer received HSCT for different types of cancer and with varied levels of disease, thus the heterogeneity of the samples further reduce likelihood of consistent findings across studies. Long-term HRQOL also should be compared between childhood cancer survivors who received allogeneic vs. autologous HSCT. Encouragingly, five of the studies in this present review were multicenter studies, and six of the studies used the same HRQOL measurement (SF-36), enabling comparisons between the studies. Larger longitudinal studies are needed to confirm these findings in medically and demographically diverse samples. We emphasize the need for prospective longer-term studies of late effects and HRQOL following childhood HSCT. Risk factors related to the development of chronic pain require more study. Interventions to prevent chronic health conditions and reduce cancer-related pain must be developed. Studies are needed in the prevention of poor HRQOL during treatment, as well as interventions for those survivors with compromised HRQOL.

## Conclusion

While many survivors of childhood HSCT enjoy good HRQOL, those with a severe chronic health condition or a major medical condition, GVHD, or pain appear to have the greatest association with poor HRQOL. Survivors of childhood HSCT require on-going, life-long monitoring as many late adverse effects may not become manifest for years or even decades after transplantation. HRQOL issues should be discussed with patients, and survivors should be informed about possible late effects to enhance monitoring and prevention. Interventions addressing HRQOL in this survivor population should become a priority. Long-term longitudinal studies should be emphasized in future research in childhood HSCT survivors, including a specific focus on pain.

## Author contributions

TR: Data search, reading and categorization of the included articles, drafting and revising the manuscript. Agreed to be accountable for the content of the work. MT: Reading and categorization of the included articles, drafting and revising the manuscript. Agreed to be accountable for the content of the work. LZ: Data search. Drafting and revising the manuscript, final approval. Agreed to be accountable for the content of the work.

### Conflict of interest statement

The authors declare that the research was conducted in the absence of any commercial or financial relationships that could be construed as a potential conflict of interest.
